# The Effect of Deflections and Elastic Deformations on Geometrical Deviation and Shape Profile Measurements of Large Crankshafts with Uncontrolled Supports

**DOI:** 10.3390/s20195714

**Published:** 2020-10-08

**Authors:** Krzysztof Nozdrzykowski, Stanisław Adamczak, Zenon Grządziel, Paweł Dunaj

**Affiliations:** 1 Faculty of Marine Engineering, Maritime University of Szczecin, 1-2 Wały Chrobrego St., 70-500 Szczecin, Poland; k.nozdrzykowski@am.szczecin.pl (K.N.); z.grzadziel@am.szczecin.pl (Z.G.); 2 Faculty of Mechatronics and Mechanical Engineering, Kielce University of Technology, Kielce, 7 Tysiąclecia Państwa Polskiego Ave., 25-314 Kielce, Poland; adamczak@tu.kielce.pl; 3 Faculty of Mechanical Engineering and Mechatronics, West Pomeranian University of Technology, 19 Piastów Ave., 70-310 Szczecin, Poland

**Keywords:** crankshaft, geometrical error, finite element model, eccentricity, Fourier series, discrete Fourier transform

## Abstract

This article presents a multi-criteria analysis of the errors that may occur while measuring the geometric deviations of crankshafts that require multi-point support. The analysis included in the paper confirmed that the currently used conventional support method—in which the journals of large crankshafts rest on a set of fixed rigid vee-blocks—significantly limits the detectability of their geometric deviations, especially those of the main journal axes’ positions. Insights for performing practical measurements, which will improve measurement procedures and increase measurement accuracy, are provided. The results are presented both graphically and as discrete amplitude spectra to make a visual, qualitative comparison, which is complemented by a quantitative assessment based on correlation analysis.

## 1. Introduction

Methodological errors occur when a model fails to include the factors of the measurement method and related phenomena. This quantity is the discrepancy between the model characteristics of the method and its real characteristics. The accuracy of measurements requires for the possible errors to be analyzed and eliminated as much as possible or treated as correction factors, especially for measurements where analysis will significantly affect the total method error. Examining and analyzing individual error components facilitates the determining of how they influence the measurement accuracy [1]. Taking these errors into account during measurements creates a wide range of possibilities for using the developed method in practical measurements.

Specific procedures for measuring large machinery components have been discussed in several studies [2,3,4]. They include methods to measure geometrical deviations of cylindrical surfaces of such components and include a concrete, broadly understood analysis of systematic and random errors in the proposed methods [5,6,7,8]. These studies contain valuable information for understanding the discussed issues and for perfectly matching modern metrology trends [9].

The error analysis elaborated in [2,10,11,12] is particularly useful for issues related to a specific group of large machine components with cylindrical assemblies [13], such as large crankshafts of ship engines. Such shafts have large masses and dimensions, and are also flaccid, have low and variable rigidity, and are susceptible to flexural deformation [14,15]. These properties require the main journals to be supported at multiple points in a controlled manner during measurements [16,17]. 

As shown in [10], the reaction forces at the interface of the main journals and supports vary along the shaft and also depend on the shaft rotation angle at the supports. An uncontrolled support—when a crankshaft’s main journals are borne by a set of fixed, rigid vee-blocks [18,19] or when any of the main journals is not supported—causes shaft deflections that cannot be eliminated. Importantly, the supports should not limit the possible journal movements, which occur when the journal axes are not mutually aligned. Misalignment occurs when the shape and geometry of manufactured items deviate from their theoretical designs, and such geometrical quantities should be correctly characterized. If shaft journals are supported by a set of fixed, rigid vee-blocks, this type of displacement is limited by unintentional preliminary deflections, resulting in incorrect measurements of geometric quantities [11]. 

Therefore, to accurately assess the crankshaft geometry, measurements must be performed by controlling the reaction forces at the supports, which must be articulated and susceptible, i.e., adaptable to possible mutual displacements of the main journals due to geometric deviations of the item being measured.

The aspect related to measurement inaccuracies caused by shaft deflection under its own weight was investigated in our previous study [10], in which we described an innovative method for eliminating deformation in large crankshafts during measurement of their geometric condition. The method consists of using the measuring system with active compensation for shaft deflection, by means of actuators cooperating with force transducers monitoring the deflection of individual crank journals of a crankshaft being measured. The results have shown that the system is able to effectively eliminate the deflection and elastic deformation of the crankshaft under the influence of its own weight.

The continuation of the study presented in [10] was [12]. In this study, the support reaction forces were changed to minimize the crankshaft elastic deflection as a function of the crank angle. The changes of these reaction forces were determined according to the developed algorithm. The algorithm uses a mathematical model that interpolates the values of forces calculated previously with finite element software. The supports are continuously adjusted when the shaft rotates by precision current-controlled valves that operate in feedback with the force sensors measuring the actual force at the contact of support heads and main journals. 

The latest study [11] describes the use of temporary counterweights during large crankshaft measurements and presents the specifications of the measurement system and method to stabilize the forces at the supports that fix the shaft during measurements. The study showed that the proposed solution provided constant reaction forces and ensured nearly zero deflection at the supported main journals of a shaft during its rotation (during its geometry measurement).

In this paper, we investigated the effect of elastic deformations on geometrical deviation and shape profile measurements of large crankshafts with uncontrolled supports. We considered the influence of the difference in the height of the supports and the influence of the journal eccentricity on the measurement results of the shaft geometry. The main motivation of this study was to indicate the limitation of the rigid vee-blocks measuring method. The results presented in this paper confirm that the currently used conventional support method—in which the main journals of a shaft are supported by a set of fixed, rigid vee-blocks—significantly limits the detectability of geometric deviations, especially those of the journal and pin axes’ positions. The results of this study also provide insights to be considered during measurements, thereby improving the measurement procedures and increasing measurement accuracy.

The structure of the article is as follows: in Section 2, the methods of fixing crankshafts to measure geometrical deviations are presented and their limitations are indicated. Next, a study plan is formulated in order to prove that the currently used measurement methods limit the detectability of geometric deviations. According to the study plan, a finite element model of an exemplary shaft was built. Based on the finite element model, the necessary calculations were carried out, the results of which are presented in Section 3, which also includes a discussion. The main conclusions are presented in Section 4.

## 2. Materials and Methods

### 2.1. Methods of Fixing Crankshafts to Measure Geometrical Deviations

**Figure 1:**
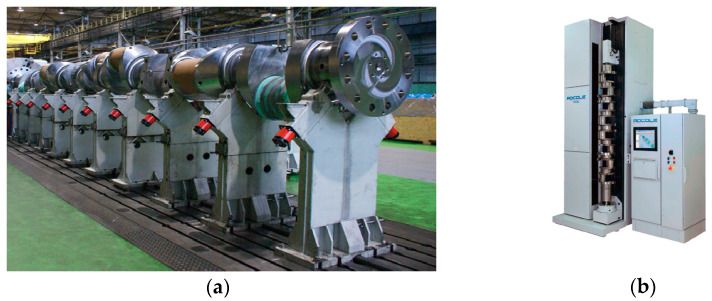
Geometrical deviation measurement methods: (**a**) horizontal on a set of rigid vee-block supports, (**b**) vertical using precision measuring machines (courtesy of ADCOLE).

As part of the methods and techniques currently used, measurements are performed with the shaft axis fixed in the horizontal or vertical plane. Large crankshafts are placed in the horizontal plane because of their large masses and dimensions. The main journals of those shafts are rested on a set of rigid vee-block supports (Figure 1a), but these conditions do not ensure the elimination of shaft elastic deflections [10,20,21]. Small and medium shafts are usually measured using precision measuring machines [22], and their axis is located in the vertical plane (Figure 1b). Those shafts are fixed and stabilized at their ends in holders or centers without additional stiffening in their middle part. With this type of stabilization, the shaft axis buckles. Both types of stabilization cause elastic deformations of the shaft that vary in sign and value due to changes in the shaft’s rigidity when it rotates.

For a horizontal shaft axis with equally elevated vee-blocks supporting all the main journals and a perfectly manufactured crankshaft (no geometrical deviations), the shaft does not undergo elastic deformations; however, the actual manufacture of machine parts always deviates from the ideal shape. Therefore, we must assume that a shaft will have geometric deviations, which qualify such shafts as usable for operation if they are within the engineering limits. Even when the geometrical deviations are within permissible limits, they cause elastic deformations of the shaft, which directly affects geometrical quantity measurements.

### 2.2. Tested Object

**Figure 2:**
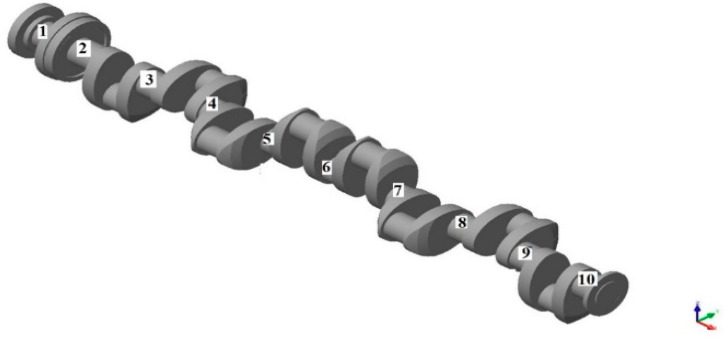
Geometrical model of the analyzed shaft with journal numeration.

The object subjected to the analysis was the crankshaft of the main propulsion medium-speed Buckau Wolf R8 DV-136 engine (Maschinenfabrik Buckau R. Wolf AG, Magdeburg, Germany), measuring 3630 mm in length and weighing 9280 N, equipped with ten 149 mm main journals and eight 144 mm crankpins. The geometrical model with main journal numeration used in further analysis is shown in Figure 2.

It was assumed that the material of which the shaft was made is AISI 1060-2 steel, characterized by Young modulus E=212 GPa, Poisson’s ratio ν=0.29, and mass density $\rho_{s}=7.7\cdot 10^{-6}\;\frac{\mathrm{kg}}{m^{3}}$.

### 2.3. Research Plan of Crankshaft Measurements

A study plan was developed in order to prove that the currently used methods of measuring crankshaft geometrical deviations, briefly described in Section 2.1, limit the detectability of geometric deviations. It includes an analysis of deflection and reaction forces at the contact of vee-block support heads with the main journals. Four most representative cases are included in the analysis, i.e.:

- Case 1: individual main journals are perfectly coaxial, while one of the supports (of journal no. 5, counting from the timing gear end) is offset upwards by 0.03 mm relative to others;
- Case 2: the main journals of all crankshafts are perfectly coaxial, while one of the supports (of journal no. 5, counting from the timing gear end) is offset downwards by 0.03 mm relative to others;
- Case 3: the axis of one of the main journals (no. 5 counting from the timing gear end) is offset upwards by 0.03 mm from the others, while the supports are at set at the same height;
- Case 4: the axis of one of the main journals (no. 5, counting from the timing gear end), is offset downwards by 0.03 mm from the others, while the supports are set at the same height.

**Figure 3:**
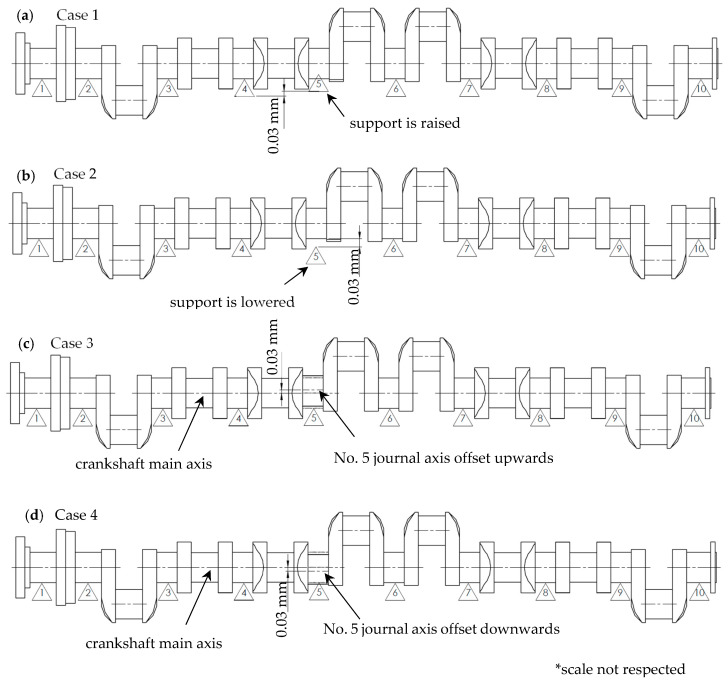
A graphic representation of the cases under consideration.

A graphic representation of the cases under consideration is shown in Figure 3.

### 2.4. Finite Element Analysis

**Figure 4:**
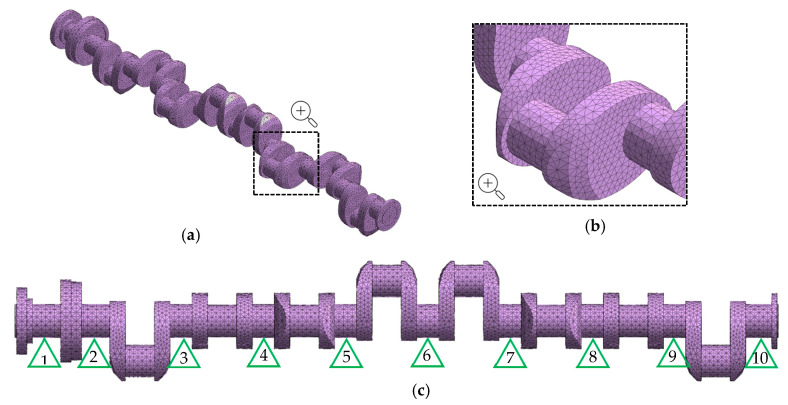
Finite element model of the analyzed shaft (**a**) isometric view; (**b**) mesh close-up; (**c**) main journals support.

To assess the deflections and reaction forces distribution at the contact between vee-block support heads with the main journals, a finite element model [23,24] of the crankshaft was established—Midas 2019 (Midas Information Technology Co. Ltd., Seongnam, Korea) [25,26]. The geometrical model of the analyzed shaft was discretized using four-sided solid elements (CTETRA) with three translational degrees of freedom in each node. As a result, the finite element model subjected to further analysis had 137,475 elements and 126,114 degrees of freedom. The finite element model is shown in Figure 4. 

The gravity load was applied to the model. An analysis consisting of determining shaft deflections and reaction forces acting on a supported shaft was performed using linear static Nastran solver (SOL101). Subsequent cases formulated in Section 2.3. were calculated according to the assumption that deformations and changes in the reaction forces on the supported main journals were caused by support positioning and geometrical deviations of the shaft when its journals were rested on a set of vee-block supports [16]. Subsequent angular positions of the shaft were simulated by rotating the model subjected to the force of gravity [27].

### 2.5. Experimental Setup

**Figure 5:**
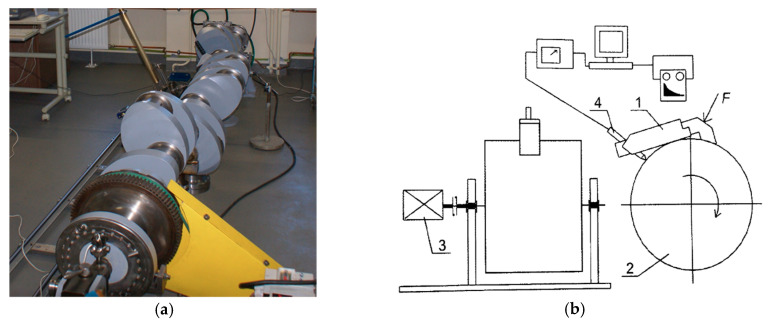
SAJD measurement system: (**a**) layout of the system; (**b**) measurement method [11]: 1—measuring head MUK 25-600, 2—shaft journal, 3—drive motor, 4—displacement sensor, F—measuring head pressing force.

Experimental measurements were performed using a constructed system consisting of a MUK 25-600 measuring head and SAJD software, which enabled a complete qualitative evaluation of the roundness profiles (Figure 5a) [2]. The MUK 25-600 head was seated directly on the surface of the journal being tested, which evaluated the shape profile independent of the object’s support conditions (Figure 5b). The roundness profile measurements were analyzed in terms of harmonics, the results of which were presented in discrete amplitude spectra [2,28].

An important advantage of this system is that measurements can be made directly in the work environment and the measured object does not need to be dismantled. Similar features have different design solutions of the measuring heads equipped with multi-contact self-adjusting vee blocks cooperating with one or more dial sensors. However, with this method it is only possible to evaluate the deviations and shape profiles, which, from the perspective of performing a comprehensive evaluation of the journal geometry, provides only a partial control of the measurement accuracy. Measuring axis deviation remains difficult.

The roundness profiles were then superimposed on the displacement profile of the center of the journal moving eccentrically and the displacement profile subject to support limitations. To completely depict the issue, the measured roundness contour was repeatedly superimposed on the displacement profile limited by the support. It was moved angularly to the defined starting position. Detailed description of the experimental system was presented in [2,11].

## 3. Results and Discussion

### 3.1. Finite Element Model Analysis

#### 3.1.1. Case One

**Figure 6:**
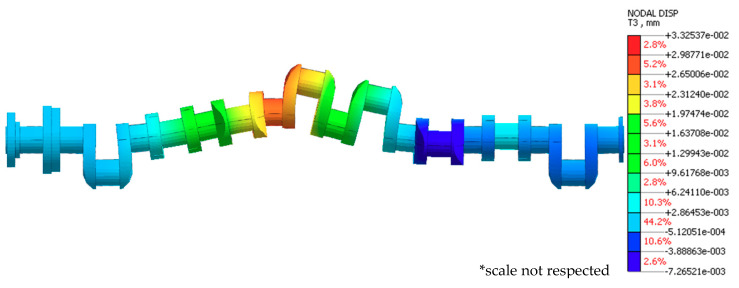
Exemplary finite element analysis results for case 1.

**Figure 7:**
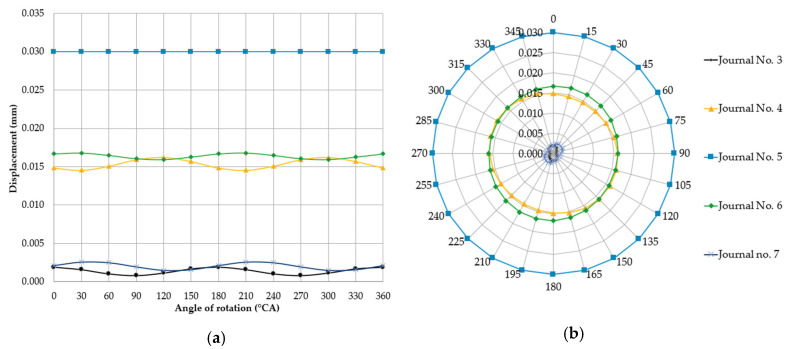
Changes in deflections measured in the vertical plane at individual main journals with the shaft rotated by 15° at a time and when one of the supports (of journal no. 5 counting from the timing gear end) is offset upwards by 0.03 mm relative to the others, shown in the charts in the (**a**) Cartesian and (**b**) polar coordinate systems.

To implement the study plan formulated in Section 2.3, we calculated the displacements and reaction forces at the contact of support heads with main journals for the support positioning and geometric shaft deviations adopted above. The positioning of the supports at different heights generally means that the shaft will be pre-deflected, even if it is perfectly constructed. It was assumed that support no. five was offset upwards by 0.03 mm relative to the others. An exemplary finite element analysis results for case one is shown in Figure 6. Figure 7 shows the graphical interpretation of the changes in deflection values for this case when changing the shaft rotation angle by 15° at a time. The displacement of the journals not included in Figure 5 was 0 mm.

For this type of support and a shaft rotation angle of 90°, Table 1 presents changes in the reaction forces at the support head/individual main journal interface.

In this case, the shaft is cyclically bent upwards and support no. five carries very high loads, completely relieving supports no. three, four, six, and seven. As support no. five is offset, the shaft is lifted, and the aforementioned supports lose contact with the journals.

#### 3.1.2. Case Two

**Figure 8:**
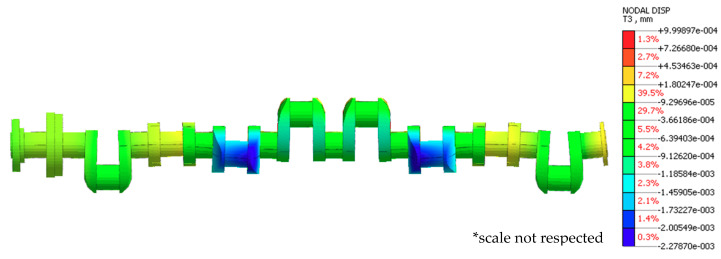
Exemplary finite element analysis results for case 2.

**Figure 9:**
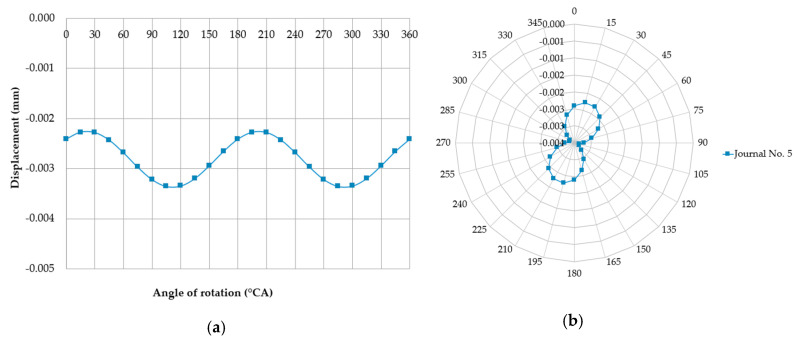
Changes in deflections measured in the vertical plane at individual main journals with the shaft rotated by 15° at a time and when one of the supports (of journal no. 5 counting from the timing gear end) was offset downwards by 0.03 mm relative to the others, in the (**a**) Cartesian and (**b**) polar coordinate systems.

Lowering the support relative to the others removes the support from under the shaft at its location. It was assumed that support no. five was offset downwards by 0.03 mm relative to the others. The results from exemplary finite element analysis for case two are shown in Figure 8. Figure 9 shows a graphical interpretation of the changes in deflection values for this case, when the shaft rotation angle was changed by 15° at a time. The displacement of the journals not included in Figure 9 was 0 mm.

Table 2 presents the changes in reaction forces at individual main journals for this type of support at a 90° shaft rotation.

The shaft bends down under its own weight in locations without support. Deflections at non-supported journals are insignificant (−0.00228 mm to −0.00335 mm), while the reaction forces at journals adjacent to the non-supported ones increase significantly, reaching 1713 N to 1773 N in journal no. four and 1541 N to 1749.9 N in journal no. six.

#### 3.1.3. Case Three

**Figure 10:**
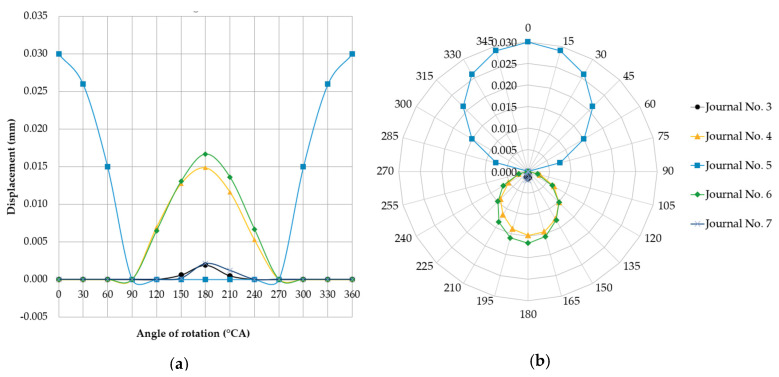
Changes in deflections measured in the vertical plane at individual main journals with the shaft changed by 15° at a time, and when one of the main journal axes (of journal no. 5 counting from the timing gear end) was offset eccentrically upwards by 0.03 mm relative to the others, shown in the (**a**) Cartesian and (**b**) polar coordinate systems.

The situation is slightly different when the supports are at the same height and the main journal axes positions deviate (case three and the alternative version of case four). In case three, the supports are located at the same height, while the axis of journal no. five (for the shaft’s reference angular position) is offset eccentrically upwards by +0.03 mm relative to the other journals. The form of deformation of the shaft is analogous to that presented in the Figure 8. Figure 10 shows a graphical interpretation of the changes in deflection values for this case, when the shaft rotation angle was changed 15° at a time. The displacement of the journals not included in Figure 10 was 0 mm.

Table 3 shows the reaction forces exerted by the supports for the four characteristic angular positions of the shaft in this case.

#### 3.1.4. Case Four

**Figure 11:**
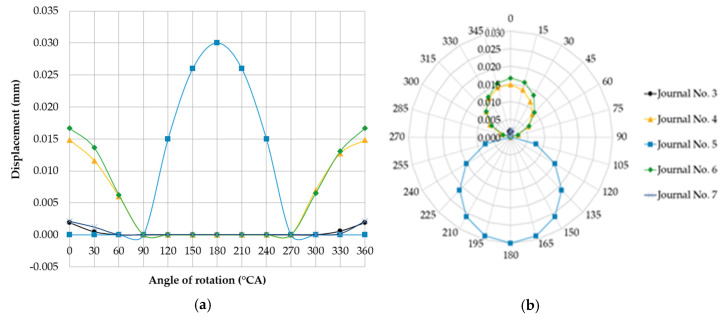
Changes in deflections measured in the vertical plane at individual main journals, when one of the main journal axes (no. 5 counting from the timing gear end) was offset eccentrically downwards by 0.03 mm relative to the others, shown in the (**a**) Cartesian and (**b**) polar coordinate systems.

Changing the shaft rotation angle by 180° causes the eccentrically located journal axis to move to an extreme location opposite the reference angle used in the previous case. The considered relative positions of the journals correspond to case four and are an alternative version of case three (Figure 8.) Using the values of deflections and reaction forces for shaft rotation angles ranging from 0° to 360°, the resulting calculated quantities will take the same values as in Table 3 if an angular offset of 180° is applied. The form of deformation of the shaft is analogous to that presented in Figure 6. Figure 11 shows a graphical interpretation of the changes in deflection values for this case, when the shaft rotation angle was changed 15° at a time. The displacement of the journals not included in Figure 11 was 0 mm. 

### 3.2. Phenomenological Model of Detectability of Journals’ Misalignment

A detailed analysis of cases three and four shows that for deviations in the position of main journal axes, the detectability of journals’ misalignment was limited by supporting the shaft with a set of fixed rigid vee-blocks located at the same height. The analysis of the graphs of journal axes’ deflections (Figure 7 and Figure 8) shows that the deflections were zero in the range of angles for which journal no. five permanently contacted the support. 

**Figure 12:**
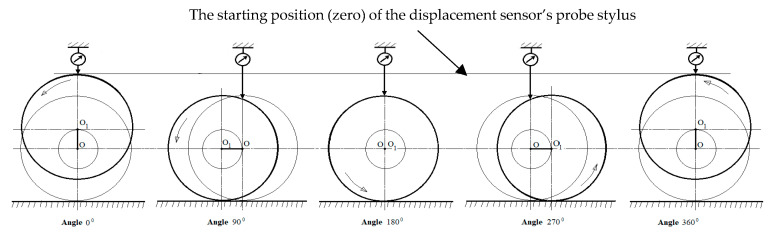
Supplementary diagram of the successive stages of shaft deflection caused by its eccentricity with respect to the axis of rotation of the measuring system when the support retains its fixed height.

We analyzed the measurements of case three, in which all the main journals of the shaft were rested on equally elevated supports, and all main journals were situated coaxially, except for journal no. five, whose axis *O*_1_ was offset upwards relative to the others by 0.03 mm in the initial angular shaft position. In case three, when the shaft was rotated in the angular range from 0 to 90°, journal no. five was unsupported, and the axis of this journal moved eccentrically with respect to the main axis *O* of the shaft (relative to the shaft’s axis of rotation). The displacement sensor, whose probe stylus is located vertically, measures the deflection of the journal resulting from its eccentric movement during the shaft rotation (Figure 12).

**Figure 13:**
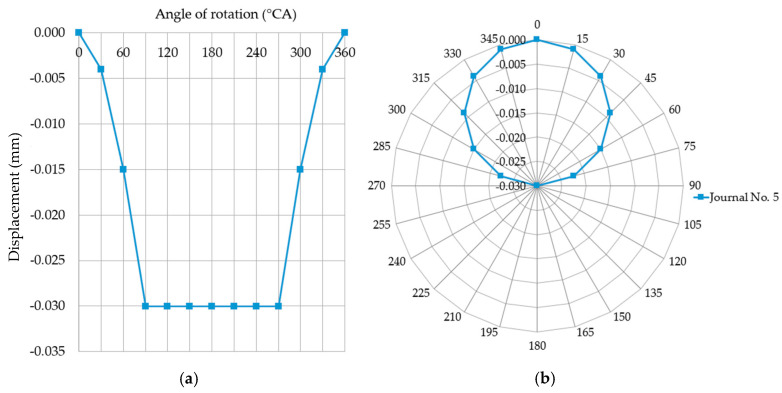
The journal displacements measured by the sensor for a full shaft rotation (0°–360° angle) are recorded when the support maintaining a constant height restricts these displacements, as shown in the (**a**) Cartesian and (**b**) polar coordinate systems.

If the dial indicator is zeroed in the top dead-center of journal no. five, and if the downward movement of the probe stylus is treated as a negative indication, then in the 90° angular position of the shaft the sensor indicator displays a value of −0.03 mm. For this angle of rotation, journal no. five will contact the support and, as the shaft rotates further (in the angular range of 90° to 180°), the reaction force will gradually increase at the point where journal no. five and the support get into contact. Simultaneously, journals adjacent to journal no. five (i.e., journals no. four, three, six, and seven) will be lifted upwards, losing contact with their supports due to bending of the shaft (caused by increased pressure of journal no. five on the support). Journal no. five is permanently in contact with its support, so the sensor will indicate a constant deflection of 0.03 mm, the same as for the angle of 90°. At shaft angles ranging from 180° to 270°, journal no. five will remain in contact with the support, which will exert gradually decreasing reaction forces on the journal. At the same time, the shaft becomes less bent at the locations of journals no. four, three, six, and seven. At 270° rotation, journal no. five loses contact with its support, and journals no. four, three, six, and seven rest on their supports. For shaft angles ranging from 180° to 270°, the displacement sensor still shows a constant deflection of –0.03 mm. For shaft rotation angles from 270° to 360°, journal no. five is gradually lifted upwards, and the displacement sensor indicates a change from –0.03 mm (at 270°) to 0.00 (at 360°). Figure 13 shows the displacements indicated by the sensor for journal no. five for shaft rotation angles from 0° to 360°.

**Figure 14:**
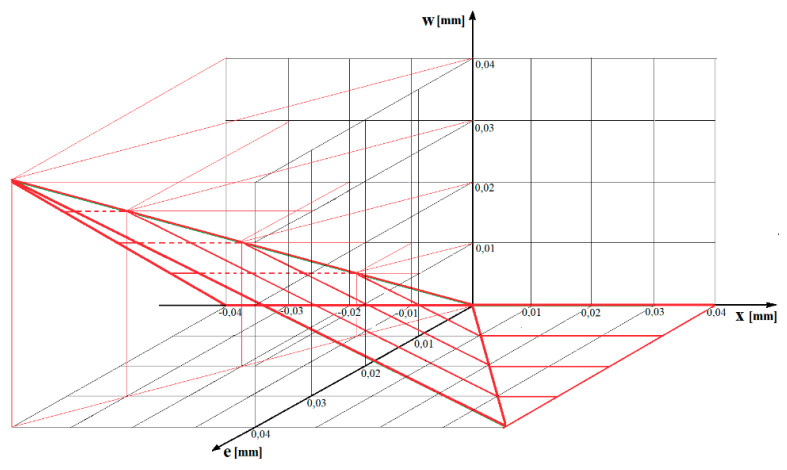
Graph showing the measurable value ***w*** of eccentricity e as a function of the vertical position x of the support.

It can be seen that in general the actual value of eccentricity can be measured by the value of this deviation and the vertical location of the support in relation to the supported main journal. Using the results obtained, a supplementary graph was drawn to show the measurable value ***w*** of eccentricity e as a function of the vertical position ***x*** of the support (Figure 14).

**Figure 15:**
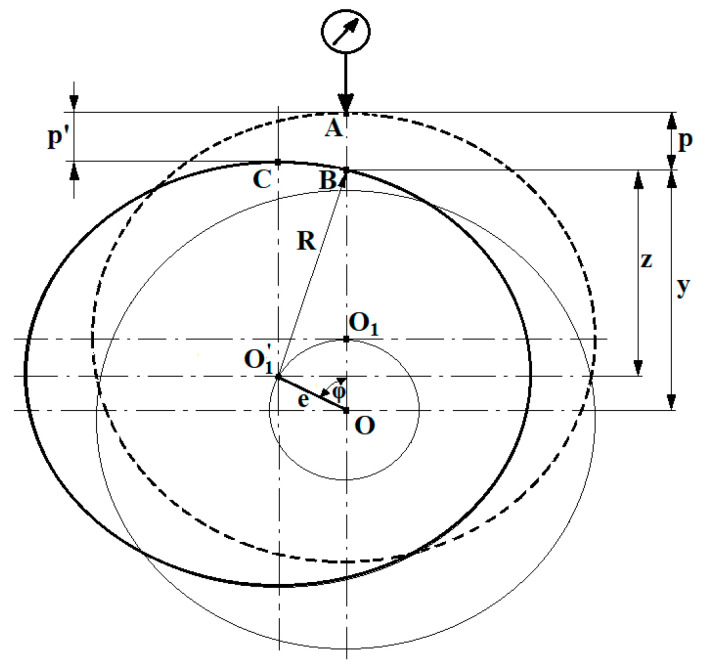
A supplementary diagram to determine the measurement error caused by a journal’s eccentric displacement when measuring geometric deviations of the shaft.

As results from the previous analysis, another issue to be considered is periodic non-overlap between the displacement direction of the sensor’s probe stylus and the axis of the journal being measured. This is caused by the eccentric movement of this axis during shaft rotation. As shown in Figure 15, for a given angular position of the shaft (angle φ), the quantity being measured is p, whereas the quantity that should be measured is $p^{\prime }$. 

By analyzing the geometrical and trigonometric relationships shown in the supplementary diagram (Figure 15), we can find a mathematical relationship that describes the measured value of p and the measurement error, i.e., the difference between p and $p^{\prime }$. According to the following diagram:

(1) $$p^{\prime }\;=\;e\;-\;e\;cos\;\varphi$$

Since

(2) y = z + e cos φ

Whereas:

(3) $$z=\;\sqrt{R^{2}-{\left(\;e\sin\varphi \right)}^{2}}$$

Thus, the final form is:

(4) $$p\;=\;R\;+\;e\;-\;y\;=\;R\;+\;e\;-\;\left(\;\sqrt{R^{2}-{\left(\;esin\varphi \right)}^{2}}+ecos\varphi \right)$$

And

(5) $$\Delta p\;=\;p\;-\;p^{\prime }\;=\;R\;-\sqrt{R^{2}-{\left(\;esin\varphi \right)}^{2}}$$

Due to the deformation, the axis of the object being measured (supported by vee-blocks) takes the angular position ϑ with respect to the probe stylus of the sensor [1,2].

**Figure 16:**
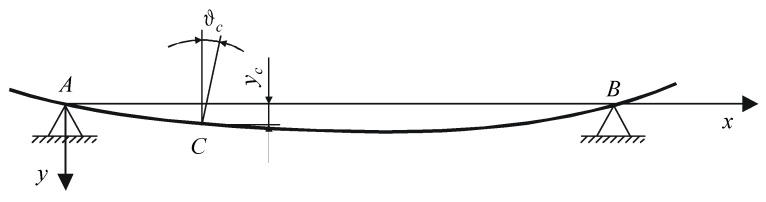
Deformation of the object supported by two vee-blocks and the linear and angular displacements resulting from this deformation.

Thus, considering the location of the section to be measured relative to the support points, the location of the center of the section being measured moves with respect to the axis of rotation (determined by the measuring system) by the value of the elastic (f) or permanent (y) deformation (Figure 16). Consequently, the measured profile of roundness is distorted by the so-called apparent eccentricity and ovality. If it is possible to determine the angular deformation *ϑ* and the arrow of deformations f or y (e.g., from strength calculations or measurements), the resulting measurement errors are systematic errors and should be used as correction factors when evaluating the proper first and second harmonic (after expanding the measurements of the roundness profile into a Fourier series [28]).

### 3.3. Journal Position Misalignment Taking into Account Eccentricity

In the considerations presented so far, it has been assumed that journals have an ideal circular profile, but machining processes involve unavoidable errors that give journals irregular roundness profiles. In general, when the shaft is fixed on vee-blocks, the shape and axial position deviations are measured in individual cross-sections of the main journals of the rotating crankshaft. In the case of a misaligned journal position, the center of the measured journal’s profile may move relative to the axis of rotation determined by the measuring system. In this case, the measurements describe the shape profile of the given cross-section, as well as the eccentricity that represents the profile center position of the section measured relative to the axis of rotation determined by the measuring system. Taking into account that a rigid support limits the detectability of geometric deviations in the main journal axes of a crankshaft (which has been demonstrated), an analysis was conducted to determine how the limited detectability of axis position deviations affects the evaluation of the main journal’s roundness profile. To accomplish this, the deviations and shape profiles of the main journals were measured for the tested crankshaft. 

**Figure 17:**
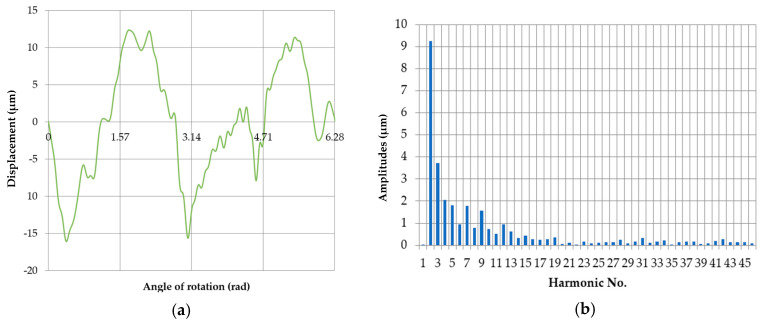
Roundness profile of journal no. 5 measured by the reference method (**a**); the discrete amplitude spectrum (**b**).

Figure 17 shows an example of a roundness profile measured by the reference method (with a MUK 25-600 sampling cell) corresponding to the roundness contour of pin no. five and a discrete amplitude spectrum obtained from the harmonic analysis. Table 4 shows the values of the individual harmonics.

**Figure 18:**
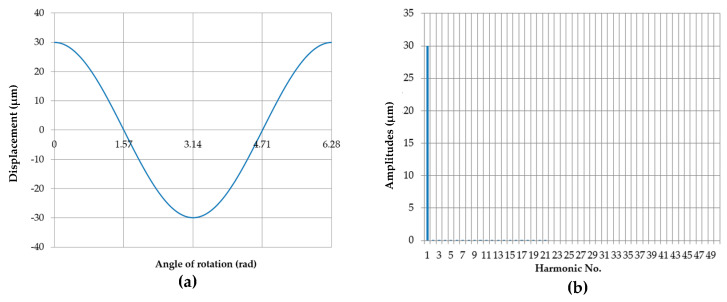
Eccentric movement profile of the center of the roundness profile being measured. The eccentricity was 0.03 mm, and the support did not limit the shaft displacement (**a**); discrete amplitude spectrum (**b**).

The image of the eccentric profile of the measured roundness contour center at an eccentricity *e* = 0.03 mm, assuming that the support does not limit the shaft displacement. The corresponding discrete amplitude spectrum is shown in Figure 18, which includes only the first harmonic (Table 5).

**Figure 19:**
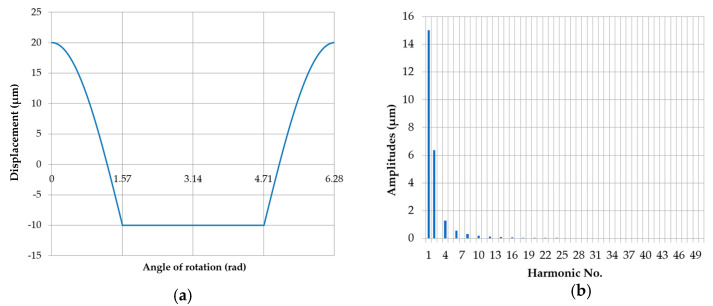
Eccentric movement profile of the center of the roundness profile being measured when the supports were set at the same height (x = 0 mm), with an eccentricity of the main journal *e* = 0.03 mm (**a**); discrete amplitude spectrum (**b**).

The image of the profile corresponding to the eccentric displacement of the center of the measured roundness profile, presented in the Cartesian system for the case when all the supports were situated at the same height (x = 0 mm), with the eccentricity of one of the main pins equal to e = 0.03 mm and the discrete amplitude spectrum is shown in Figure 19. As can be seen, the amplitude spectrum contains only even harmonic components, the values of which are summarized in Table 6.

**Figure 20:**
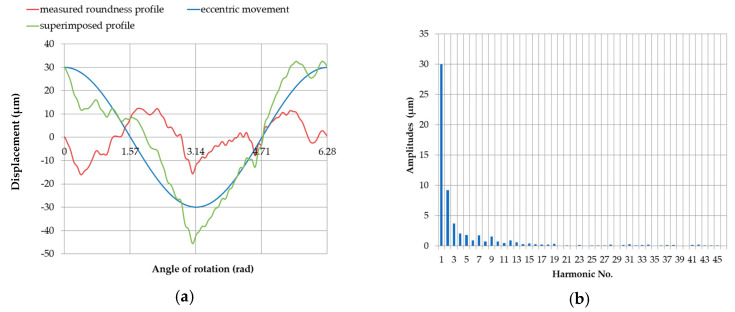
The profile obtained by superimposing the measured roundness profile of journal no. 5 on the full profile of the eccentric movement of the measured roundness profile center (**a**); discrete amplitude spectrum of the superimposed profile (**b**).

When the measured round contour was superimposed on the displacement profile of the pin center without being limited by the support, the total profile and the discrete spectrum shown in Figure 20 was obtained. The corresponding amplitude values are shown in Table 7.

**Figure 21:**
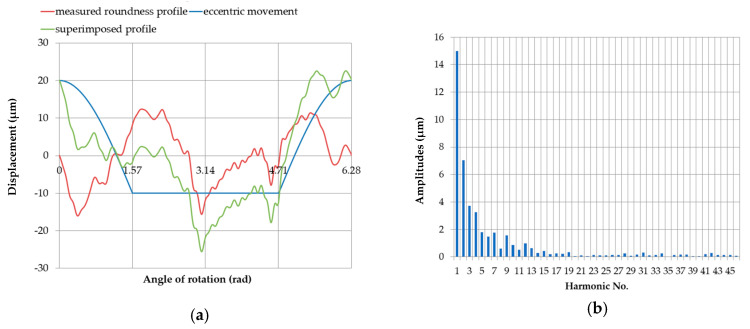
Profile obtained by superimposing the measured roundness profile of journal no. 5 on the eccentric movement profile of the center of the measured roundness profile with the supports set at the same height (x = 0 mm), with an eccentricity of the main journal *e* = 0.03 mm (**a**); discrete amplitude spectrum of the superimposed profile (**b**).

When the measured round contour was superimposed on the displacement profile of the pin center at the support limit, the total profile was obtained, which is shown in Figure 21 for the starting position. The amplitude values are shown in Table 8.

**Figure 22:**
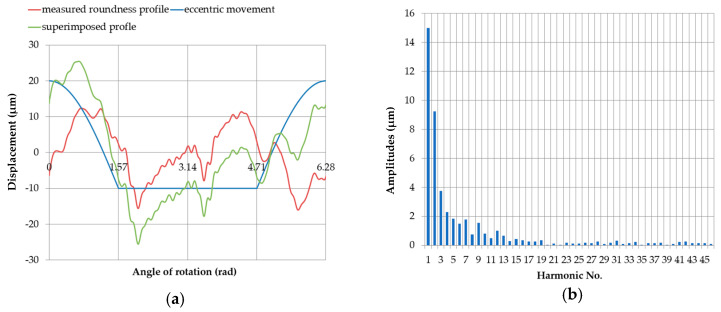
Profile obtained by superimposing the measured roundness profile of journal no. 5—rotated by 60° with respect to the reference profile—onto the profile of the eccentric movement of the center of the measured roundness profile, with the supports set at the same height (x = 0 mm); with an eccentricity of the main journal e = 0.03 mm (**a**); discrete amplitude spectrum of the superimposed profile (**b**).

**Figure 23:**
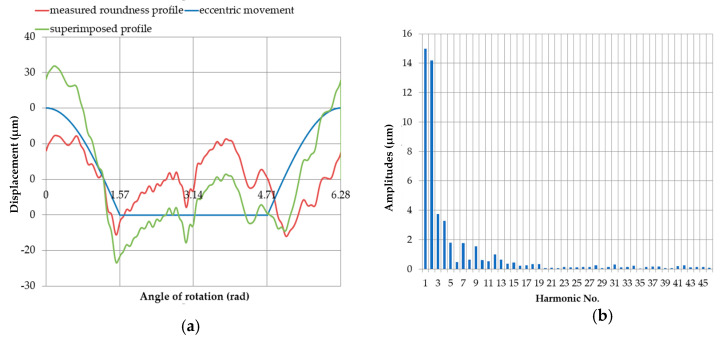
Profile obtained by superimposing the measured roundness profile of journal no. 5 (green)—rotated by 90° with respect to the reference profile—onto the eccentric movement profile of the center of the measured roundness profile (blue), with the supports set at the same height (*x* = 0 mm), with an eccentricity of the main journal e = 0.03 mm (**a**); discrete amplitude spectrum of the superimposed profile (**b**).

The image of the profile obtained after superimposition and shifting by 60°, followed by 90° (relative to the assumed starting point) of the measured roundness contour of pin no. 5, on the simultaneously displayed profile of the eccentric movement at the measured roundness contour center limited by the support are shown in Figure 22 and Figure 23, respectively. The amplitudes of the individual harmonics are shown in Table 9 and Table 10.

According to the accepted interpretation of the measured round contour geometrical features of the analysis based on harmonics, the first term in the Fourier series of the function characterizing the course is the deviation of the axis position, namely the eccentricity. Eliminating this harmonic makes it possible to treat the sum of the remaining harmonics as the theoretically measured roundness contour. 

**Figure 24:**
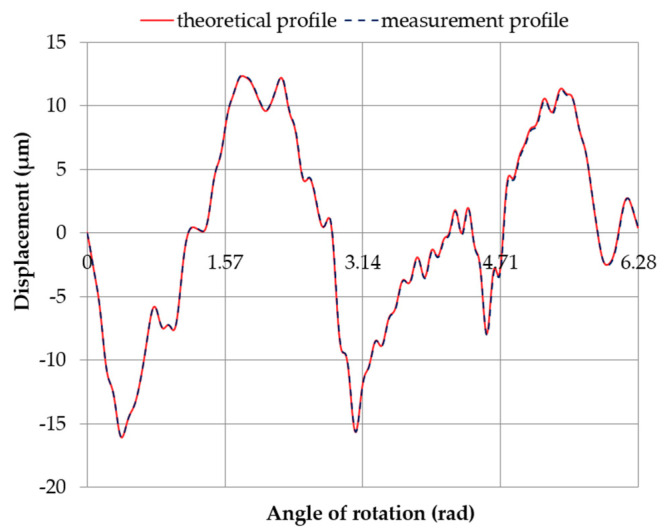
Measured and theoretical profiles (excluding harmonic component no. 1) obtained by superimposing the measured roundness profile of journal no. 5 onto the full eccentric movement profile of the center of the measured roundness profile.

**Figure 25:**
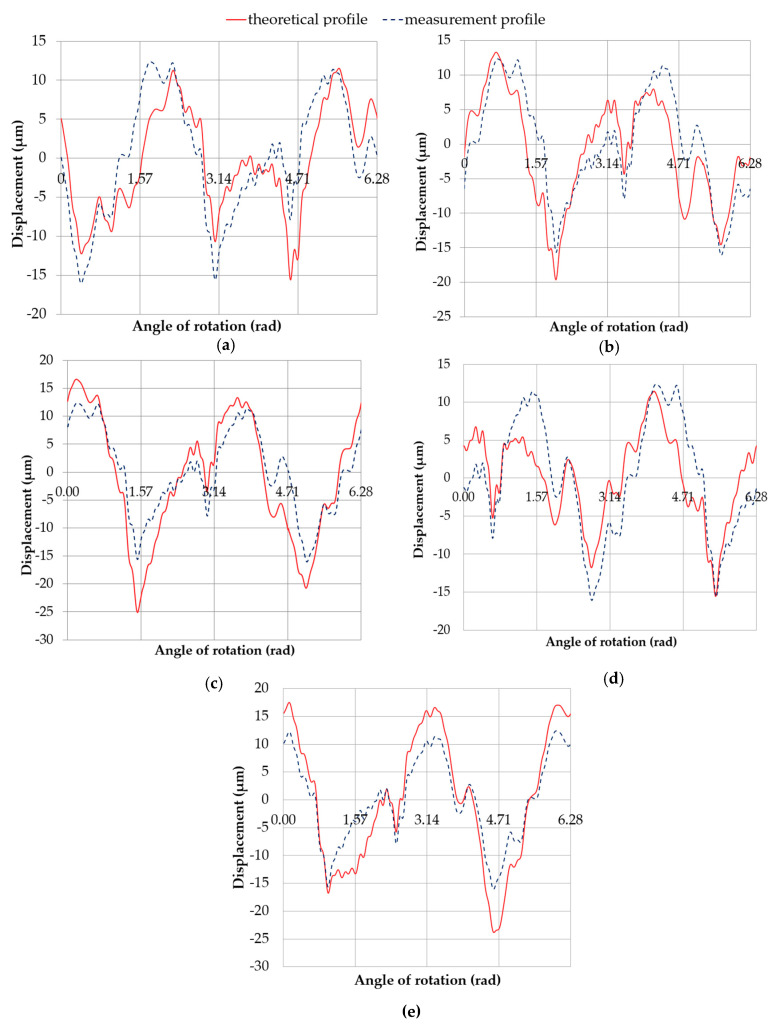
Measured and theoretical profiles (excluding harmonic component no. 1) obtained by superimposing the measured roundness profile of journal no. 5 onto the eccentric movement profile of the center of the measured roundness profile with the supports set at the same height (*x* = 0 mm), with an eccentricity of the main journal *e* = 0.03 mm, (**a**) at a starting position; (**b**) rotated 60°; (**c**) rotated by 90°; (**d**) rotated by 225°; (**e**) rotated by 300°.

This interpretation was used to qualitatively and quantitatively compare the measured and theoretical (excluding harmonic no. 1) roundness contours, obtained from superimposing the measured roundness contour of pin no. 5 on the full eccentric profile and the eccentricity profile of the measured round outline center at the support limit. A graphical representation of the compared profiles of journal no. 5 (Figure 24 and Figure 25) is helpful to visually assess the profile quality and compare the amplitude spectra.

The quantitative evaluation was conducted by determining the roundness deviations between the compared profiles and finding the correlation coefficient between the profiles provided by the formula [1,2,4,41–43]:

(6) $$\rho \left(\gamma_{\phi }\right)=\frac{2\int_{0}^{2\pi }r_{1}\left(\phi \right)r_{2}\left(\phi +\gamma_{\phi }\right)d\phi }{\int_{0}^{2\pi }r_{1}{\left(\phi \right)}^{2}d\phi +\int_{0}^{2\pi }r_{2}{\left(\phi \right)}^{2}d\phi }$$

where: $r_{1}\left(\varphi \right)$—roundness profile obtained from measurements performed by the reference method. $r_{2}\left(\varphi \right)$—roundness profile obtained from measurements performed by the proposed method. $\gamma_{\phi }$—phase shift between the compared profiles. 

The adopted procedure involved repeatedly superimposing the measured roundness profile (with angular rotation) onto the displacement profile using the support to create a limitation. This approach allowed the relative angular position of the compared profiles to be determined. It can also be used to determine the maximum and minimum correlation coefficients between the actual standard profile and the theoretical profile obtained by superimposing the measured profile onto the displacement profile, as well as the roundness deviations resulting from this procedure.

**Figure 26:**
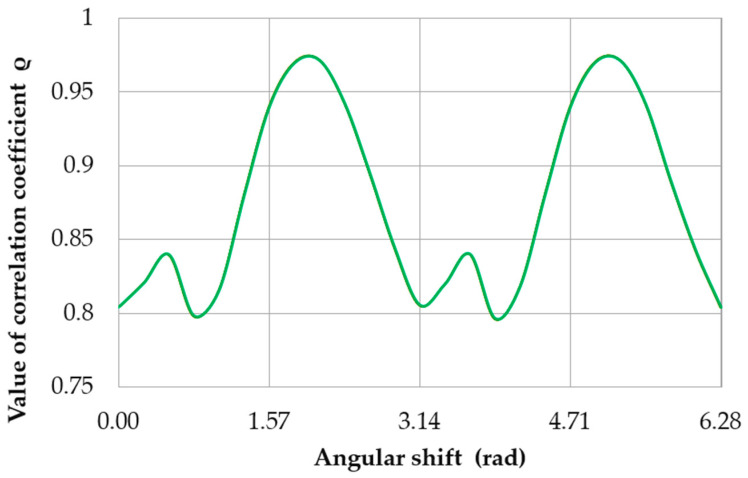
Variation in the correlation coefficient *ρ* as a function of the angular shift between the measured and evaluated profiles.

Table 11 presents the correlation coefficients ρ between the compared profiles and the roundness deviations of the evaluated profiles $\Delta_{o}$. Figure 26 shows a graph of *ρ* as a function of the angular shift between the compared profiles. The minimum correlation coefficient was $\rho_{min}=\;0.7962$, whereas the roundness deviation of the assessed profile was $\Delta_{o}\;=\;27.03\;\mu m$ (Figure 25d). The maximum correlation coefficient was $\rho_{max}=\;0.9717$, and the roundness deviation of the assessed profile was $\Delta_{o}=41.39\;\mu m$ (Figure 25e). 

## 4. Conclusions

The study presented in this article confirmed that the detectability of geometric deviations is limited when the shaft is supported in an uncontrolled manner (with a set of rigid vee-block supports), which was especially true for the main journal deviations. However, the limited detectability of large crankshafts due to the support conditions was observed for positional deviations of journal axes and also for shape profile deviations in journals. The shape deviation measurements may vary significantly in terms of their values and profiles relative to the actual shape of an object. Referring to the denotations used in the article, when a journal axis moved eccentrically as the shaft rotated, the investigated parameter values were directly influenced by the eccentricity *e*, the support location *x*, and the location of the measured journal profile reflecting the journal axis displacement. 

The results of this study show the importance of ensuring appropriate support conditions to eliminate deflections, and thus elastic deformations of the crankshaft under the influence of its own weight, as well as those caused by its geometric deviations. These deflections can only be eliminated if there is constant contact between the supports and the main journals of the shaft. Such conditions cannot be guaranteed by supporting the shaft with a set of rigid vee-block supports maintaining a fixed height. For deviations in the position of main journal axes, unintentional pre-deflections generate elastic deformations when the shaft rotates. This state causes interactions between geometrical deviations and elastic deformations (which are interrelated), and the geometric evaluation of the shaft geometry becomes unreliable.

Therefore, to ensure correct measurement conditions, the main journals of the shaft should be supported with a set of supports that compensate for its deflections and elastic deformations under the influence of its own weight, as well as those caused by geometric deviations of the shaft. The reaction forces at the contact between support heads and main journals vary along the shaft, and also depend on the angle of rotation of the shaft being supported, thereby ensuring zero deflections at the journals.

## 5. Patents

1. Nozdrzykowski, K. Device for measuring positional deviation of axis of crankshaft pivot set. Polish Patent Office, PL393829-A1; PL218653-B1.

## Figures and Tables

**Table 1:** Changes in the reaction forces at the contact of the supports with each main journal when the shaft rotation angle was changed by 90° and one of the supports (of journal no. 5 counting from the timing gear end) was offset upwards by 0.03 mm relative to the others.

Angular Position [°CA]	Main Journal Number [-]									
	1	2	3	4	5	6	7	8	9	10
	Reaction Forces in Main Journals [N]									
0°	681.2	1545.4	0	0	3891.3	0	0	1441.7	1157.6	566.5
90°	878.8	1302.2	0	0	3928.8	0	0	1470.9	1162.4	540.7
180°	681.2	1545.4	0	0	3891.3	0	0	1441.7	1157.6	566.5
270°	878.8	1302.2	0	0	3928.8	0	0	1470.9	1162.4	540.7

**Table 2:** Changes in the reaction forces where the supports contact each main journal when the shaft rotation angle was changed by 90° with one of the supports (of journal no. 5 counting from the timing gear end) offset downwards by 0.03 mm relative to the others.

Angular Position [°CA]	Main Journal Number [-]									
	1	2	3	4	5	6	7	8	9	10
	Reaction Forces in Main Journals [N]									
0°	725.4	996.9	729.4	1714.0	0	1750.0	584.3	1183.4	1025.2	572.3
90°	817.3	844.7	775.5	1773.6	0	1541.2	914.9	907.9	1168.4	540.3
180°	725.4	996.9	729.4	1714.0	0	1750.0	584.3	1183.4	1025.2	575.3
270°	817.3	844.7	775.5	1773.6	0	1541.2	914.9	907.9	1168.4	540.3

**Table 3:** Changes in the reaction forces where the supports contact each main journal when the shaft rotation angle changes by 90° and when one of the axis main journals (journal no. 5 counting from the timing gear end) is offset upwards by 0.03 mm relative to the others.

Angular Position [°CA]	Main Journal Number [-]									
	1	2	3	4	5	6	7	8	9	10
	Reaction Forces in Main Journals [N]									
0°	725.5	996.9	729.4	1714.0	0	1750.0	584.3	1183.4	1025.2	575.3
90°	833.3	763.1	1134.1	891.3	1126.1	809.9	1127.1	886.2	1173.4	539.5
180°	681.2	1545.4	0	0	3891.3	0	0	1441.7	1157.6	566.5
270°	833.3	763.1	1134.0	891.3	1126.1	809.9	1127.1	886.2	1173.4	539.5

**Table 4:** Amplitudes of the harmonic components for the roundness profile of journal no. 5, measured by the reference method.

Harmonic Amplitudes [μm]					
n	0	10	20	30	40
n + 0		0.733112	0.048821	0.160022	0.078616
n + 1		0.519559	0.100569	0.321846	0.20063
n + 2	9.253684	0.958851	0.030333	0.117518	0.266181
n + 3	3.736699	0.637086	0.153547	0.154698	0.127993
n + 4	2.063435	0.326033	0.084396	0.233796	0.136985
n + 5	1.80228	0.441492	0.116786	0.028151	0.134468
n + 6	0.958385	0.279591	0.139388	0.128674	0.082787
n + 7	1.778264	0.254698	0.142395	0.159856	0.062551
n + 8	0.773096	0.277479	0.244795	0.176508	0.088632
n + 9	1.558816	0.344293	0.073347	0.049974	0.007096

**Table 5:** Amplitudes of the harmonic components for the eccentric movement of the center of the roundness profile being measured for eccentricity e = 0.03 mm where the support does not limit the shaft displacement.

	Harmonic Amplitudes [μm]				
n	0	10	20	30	40
n + 0		0.009154	0.002274	0.001011	0.00057
n + 1	29.99956	0.007553	0.002062	0.000947	0.000542
n + 2	0.00059	0.006339	0.001879	0.000889	0.000517
n + 3	0.000221	0.005396	0.001719	0.000836	0.000493
n + 4	0.000118	0.004649	0.001579	0.000788	0.000471
n + 5	7.37 × 10^−5^	0.004048	0.001455	0.000743	0.000451
n + 6	5.06 × 10^−5^	0.003556	0.001345	0.000703	0.000431
n + 7	3.69 × 10^−5^	0.003149	0.001248	0.000665	0.000413
n + 8	2.81 × 10^−5^	0.002808	0.00116	0.000631	0.000397
n + 9	2.21 × 10^−5^	0.00252	0.001082	0.000599	0.000381

**Table 6:** Amplitudes of the harmonic components for the eccentric movement of the center of the roundness profile being measured when the supports were set at the same height (x = 0 mm), with an eccentricity of the main journal e = 0.03 mm.

Harmonic Amplitudes [μm]					
n	0	10	20	30	40
n + 0		0.1930	0.0479	0.0213	0.0120
n + 1	14.9993	0.0001	0.0000	0.0000	0.0000
n + 2	6.3665	0.1336	0.0396	0.0187	0.0109
n + 3	0.0006	0.0001	0.0000	0.0000	0.0000
n + 4	1.2731	0.0980	0.0333	0.0166	0.0099
n + 5	0.0001	0.0001	0.0000	0.0000	0.0000
n + 6	0.5458	0.0750	0.0284	0.0148	0.0091
n + 7	0.0002	0.0000	0.0000	0.0000	0.0000
n + 8	0.3032	0.0592	0.0245	0.0133	0.0084
n + 9	0.0001	0.0001	0.0000	0.0000	0.0000

**Table 7:** Amplitudes of harmonic components for the profile obtained by superimposing the measured roundness profile of journal no. 5 on the full eccentric movement profile of the center of the measured roundness profile.

Harmonic Amplitudes (μm)					
n	0	10	20	30	40
n + 0		0.73	0.05	0.16	0.08
n + 1	30.00	0.52	0.10	0.32	0.20
n + 2	9.25	0.96	0.03	0.12	0.27
n + 3	3.74	0.64	0.15	0.15	0.13
n + 4	2.06	0.33	0.08	0.23	0.14
n + 5	1.80	0.44	0.12	0.03	0.13
n + 6	0.96	0.28	0.14	0.13	0.08
n + 7	1.78	0.25	0.14	0.16	0.06
n + 8	0.77	0.28	0.24	0.18	0.09
n + 9	1.56	0.34	0.07	0.05	0.01

**Table 8:** Amplitudes of harmonic components for the profile obtained by superimposing the measured roundness profile of journal no. 5 on the profile of the eccentric movement of the center of the measured roundness profile when the supports are set at the same height (x = 0 mm); with an eccentricity of the main journal e = 0.03 mm.

Harmonic Amplitudes [μm]					
n	0	10	20	30	40
n + 0		0.8665	0.0542	0.1725	0.0686
n + 1	14.9990	0.5196	0.1006	0.3218	0.2006
n + 2	7.0416	0.9778	0.0484	0.1178	0.2767
n + 3	3.7370	0.6371	0.1536	0.1547	0.1280
n + 4	3.2658	0.2978	0.1164	0.2504	0.1456
n + 5	1.8022	0.4414	0.1168	0.0282	0.1345
n + 6	1.4820	0.2104	0.1314	0.1350	0.0857
n + 7	1.7784	0.2547	0.1424	0.1599	0.0625
n + 8	0.6172	0.2376	0.2624	0.1841	0.0867
n + 9	1.5587	0.3442	0.0734	0.0500	0.0071

**Table 9:** Amplitudes of the harmonic components for the profile obtained by superimposing the measured roundness profile of journal no. 5—and by rotating it by 60° with respect to the reference profile—onto the eccentric movement profile of the center of the measured roundness profile when the supports were set at the same height (x = 0 mm), with an eccentricity of the main journal *e* = 0.03 mm.

Harmonic Amplitudes [μm]					
n	0	10	20	30	40
n + 0		0.7893	0.0343	0.1623	0.0844
n + 1	15.0011	0.4982	0.1035	0.3275	0.2169
n + 2	9.2514	0.9881	0.0176	0.0956	0.2657
n + 3	3.7533	0.6462	0.1620	0.1455	0.1292
n + 4	2.2880	0.2782	0.1145	0.2314	0.1355
n + 5	1.8372	0.4334	0.1085	0.0266	0.1317
n + 6	1.5002	0.3518	0.1749	0.1302	0.0869
n + 7	1.7701	0.2455	0.1344	0.1570	0.0000
n + 8	0.7415	0.2474	0.2530	0.1678	0.0000
n + 9	1.5501	0.3552	0.0730	0.0419	0.0000

**Table 10:** Amplitudes of harmonic components for the profile obtained by superimposing the measured roundness profile of journal no. 5—and rotating it by 90° with respect to the reference profile—onto the profile of the eccentric movement of the center of the measured roundness profile with the supports set at the same height (x = 0 mm), with an eccentricity of the main journal *e* = 0.03 mm.

Harmonic Amplitudes [μm]					
n	0	10	20	30	40
n + 0		0.6211	0.0587	0.1537	0.0677
n + 1	14.9993	0.5201	0.1002	0.3213	0.2007
n + 2	14.1993	0.9872	0.0539	0.1215	0.2559
n + 3	3.7376	0.6367	0.1542	0.1540	0.1275
n + 4	3.2729	0.3710	0.1142	0.2185	0.1461
n + 5	1.8030	0.4409	0.1167	0.0283	0.1351
n + 6	0.4735	0.2148	0.1557	0.1315	0.0783
n + 7	1.7790	0.2554	0.1431	0.1595	0.0000
n + 8	0.6317	0.3263	0.2645	0.1673	0.0000
n + 9	1.5596	0.3435	0.0730	0.0496	0.0000

**Table 11:** Roundness deviations Δ_o_ for the profiles compared in Figure 21 and Figure 22. The correlation coefficients *ρ* between the compared profiles.

Profile/Figure	Roundness Deviation Δ_o_ [μm]		Correlation Coefficient *ρ*
	Standard (Reference)	Evaluated	
Figure 25	28.40	28.40	0.9999
Figure 25a		26.86	0.8009
Figure 25b		32.95	0.8206
Figure 25c		41.65	0.9330
Figure 25d		27.03	0.7962
Figure 25e		41.39	0.9717
